# Adrenomedullin as a New Prosperous Biomarker in Infections: Current and Future Perspectives

**DOI:** 10.3390/jcm13206142

**Published:** 2024-10-15

**Authors:** Gabriela Trojan, Anna Moniuszko-Malinowska, Anna Grzeszczuk, Piotr Czupryna

**Affiliations:** Department of Infectious Diseases and Neuroinfections, Medical Uniwersity of Bialystok, 15-540 Bialystok, Poland; annamoniuszko@op.pl (A.M.-M.); anna.grzeszczuk@umb.edu.pl (A.G.); avalon-5@wp.pl (P.C.)

**Keywords:** MR-proADM, ADM, mid-regional proadrenomedullin, marker, adrenomedullin, infection

## Abstract

Adrenomedullin has emerged as a promising biomarker in the field of viral diseases. Numerous studies have demonstrated its potential in assessing disease severity, predicting clinical outcomes, and monitoring treatment response. Adrenomedullin (AM) is a multifaceted peptide implicated in vasodilation, hormone secretion, antimicrobial defense, cellular growth, angiogenesis, and, importantly, chronic pain. AM and related peptides interface with cytoskeletal proteins within neuronal contexts, influencing microtubule dynamics. AM has primarily been utilized in diagnosing diseases of bacterial origin, including sepsis. Nevertheless, there are reports suggesting its utility in diseases of viral origin, and this is the focus of the present study. Furthermore, adrenomedullin has been shown to be elevated in various viral infections, suggesting its role in immune response modulation. Furthermore, AM may contribute to neuronal dysfunction through mechanisms involving immune and inflammatory responses, apoptosis, and disruptions in calcium homeostasis. This review aims to consolidate current knowledge regarding AM and its potential implications in viral diseases, elucidating its diverse roles in neurological pathophysiology. This review highlights the growing importance of adrenomedullin as a biomarker in viral diseases and the need for further functional studies to understand the underlying mechanisms involved.

## 1. Introduction

There are many prospective biomarkers that could prove valuable in diagnosing, tracking, and managing viral diseases [1,2]. However, given the wide array of viruses and the intricate nature of the body’s response to infection, it is not always possible to identify a singular, universally applicable marker that is both highly specific and sensitive across all viral strains [2,3].

Viral illnesses pose a significant global health threat due to their potential for severe consequences and rapid transmission [4,5]. Emerging and re-emerging viral pathogens continue to pose a significant threat to public health, as evidenced by observations made over the past few years [5,6]. They can affect various organs and systems in the body, resulting in a wide range of symptoms and conditions, from mild infections to serious diseases like influenza, HIV/AIDS, COVID-19, and viral hepatitis [4].

Characteristic features of viral illnesses include their sudden onset of symptoms, rapid spread among populations, and genetic variability, all of which present challenges for prevention and treatment [4,5]. Addressing viral diseases requires global cooperation, swift scientific responses, and effective healthcare strategies, including vaccination, preventive measures, and pharmacological interventions, particularly with the emergence of new viruses like SARS-CoV-2 [7,8].

Additionally, due to the adaptability of viruses and the need for quick and accurate diagnosis, there is a growing demand for the exploration of innovative biomarkers for viral diseases [1,9]. The discovery of specific and sensitive markers could significantly expedite infection identification, disease progression monitoring, and treatment efficacy assessment [1,10]. Consequently, research into new biomarkers is a critical area of scientific inquiry with the potential to greatly advance the management of viral diseases and improve public health outcomes [11].

Viral meningitis and encephalitis require special attention. These illnesses have high mortality and morbidity rates and lead to severe neurological sequelae, causing long-term consequences for individual quality of life and societal well-being [12,13,14]. It is commonly observed that viruses are the predominant organisms responsible for infectious encephalitis [14].

## 2. Search Strategy

To assess the prognostic value of adrenomedullin and mid-regional proadrenomedullin (MR-proADM) in viral diseases, a comprehensive search was conducted on PubMed and Scopus from 1 November 2023 to 30 May 2024. This search was further supplemented by a Google Scholar query. Specific and effective search strategies were employed for each database utilizing the following terms: ‘MR-proADM’, ‘mid-regional proadrenomedullin’, ‘adrenomedullin’, ‘ADM’, ‘biomarkers’, ‘encephalitis’, ‘brain’, and ‘viral diseases’. The search strategy incorporated both Medical Subject Headings (MeSH) and text words. Additionally, the reference lists of relevant articles were reviewed to identify further pertinent studies. The results were further screened by title and abstract.

## 3. Eligibility Criteria

For at least one or more of the following outcomes—encephalitis, brain injury, or lack of brain function—we considered original studies and meta-analyses reporting adrenomedullin levels in patients with neurodegenerative conditions and viral infections. Only full-text studies in English and Polish were included.

**Figure 1:**
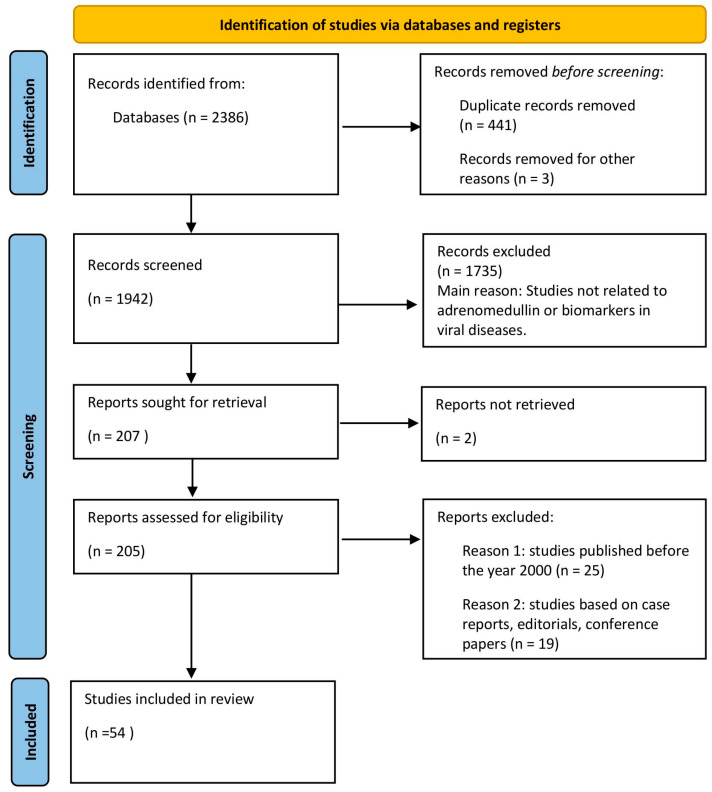
Study selection and exclusion process flowchart.

The exclusion criteria for the meta-analysis were the following: (1) studies published before the year 2000; (2) studies based on case reports, editorials, or conference papers; and (3) studies published in languages other than English and Polish. The article selection process is presented in Figure 1.

## 4. Adrenomedullin

Adrenomedullin (AM) is a peptide that belongs to the calcitonin gene-related peptide (CGRP) family, which includes CGRP, calcitonin, intermedin (IMD/AM2), amylin, and calcitonin receptor-stimulating peptide [15,16,17,18,19]. The messenger RNA (mRNA) carries the genetic instructions for synthesizing preproadrenomedullin. This precursor is then converted into proadrenomedullin, which is further divided into several components: adrenomedullin (ADM), proadrenomedullin N-terminal peptide (PAMP), adrenotensin, and mid-regional proadrenomedullin (MR-proADM) [20]. The ADM level indicates the extent of endothelial damage and correlates with the severity of organ damage observed in severe infections [21,22]. While the breakdown of endothelial barrier function is a characteristic feature of acute inflammation and contributes to the fatal organ dysfunction seen in severe infections, there is currently no adequate therapy available to stabilize endothelial barrier function [22,23]. Studies focusing on vascular models have examined the ability of CGRP and adrenomedullin to penetrate the blood–brain barrier (BBB), indicating that neither peptide can cross the BBB when the vascular endothelium remains intact [15].

A comprehensive investigation has been conducted on adrenomedullin (AM), demonstrating that it typically does not traverse the blood–brain barrier or gain access to the brain under normal circumstances [15,24].

AM potentially plays a role in preserving the integrity of the blood–brain barrier, regulating cerebral circulation and modulating the volume of cerebrovascular fluid [15]. Expression of mRNA and immunoreactivity for adrenomedullin (AM) has been observed in both human and rat brains, with localization evident in the vasculature, choroid plexus, and neurons and glial cells within the hypothalamus, cerebellum, and medulla [15,25].

MR-proADM has a longer half-life than ADM, and that is why it is more useful. Therefore, it is more frequently used in research studies [20,26]. The levels of mid-regional proadrenomedullin, originating from proADM, directly mirror the impacts of its precursor [26]. It has found applications in diseases of both bacterial and viral origin [20].

The human AM gene is expressed in numerous tissues, such as the heart, eye, adrenal gland, kidney, liver, lung, thyroid, and brain [27]. AM exerts its biological effects by binding to a receptor that consists of a heterodimer formed by the calcitonin receptor-like receptor and receptor activity-modifying proteins, RAMP2 or RAMP3, known as AM1 and AM2 receptors, respectively [15,28]. AM predominantly acts on the AM1 receptor [20,29]. Scientists agree that mid-regional proadrenomedullin demonstrates significant specificity for brain tissue [30].

Under normal circumstances, in a healthy, young individual, the level of adrenomedullin is typically very low. Factors contributing to its elevation in the body include excessive catecholamine production, hypoxia, oxidative stress (cellular), pro-inflammatory factors, and certain cytokines [20,31].

An argument in favor of MR-proADM is that such a biomarker can be readily and expeditiously assessed utilizing a fully automated platform.

Originally identified as a vasoactive peptide, AM has since been implicated in a wide range of physiological and pathological processes, including bronchodilation, the promotion of diuresis, the regulation of food intake, hormone modulation, gastrointestinal function, cell apoptosis, immune response, tumor progression, and diabetes [32,33,34]. Some sources suggest that proadrenomedullin exhibits antibacterial activity [20,35]. The role of AM in neurological diseases is increasingly being explored, with evidence indicating that AM functions as an inflammatory mediator, neuromodulator, neurotransmitter, and neurohormone through autocrine or paracrine mechanisms [20,36]. The extensive distribution of AM and its receptors throughout the nervous system underscores its significant and multifaceted role in neural function [36].

Some sources indicate that the average increase in plasma adrenomedullin levels in individuals with infectious diseases oscillates around a twofold increase compared to that in healthy individuals [18]. It is estimated that the level of adrenomedullin is higher in bacterial diseases than in viral ones [20,37,38].

## 5. Bacterial Diseases

Severe bacterial infections initiate an inflammatory response marked by increased levels of C-reactive protein and neopterin, as well as oxidative stress, which consequently elevates NO levels. This inflammation causes direct damage to endothelial cells by triggering endothelitis and upregulating adrenomedullin expression [20]. This is precisely why adrenomedullin has been utilized as a marker in diseases of bacterial origin.

## 6. Sepsis

Sepsis emerges as a consequence of an aberrant immune reaction to infection, culminating in severe organ dysfunction [39]. Together with septic shock, it constitutes a significant peril to the lives of critically ill patients. On a global scale, sepsis affects upward of 30 million individuals each year, contributing to an estimated 6 million fatalities annually [40].

Endothelial dysfunction, a critical consequence of SARS-CoV-2 infection, is centrally involved in causing damage to several organs and significantly increases the likelihood of death [26,41,42]. Only a limited set of biomarkers and a handful of commercially accessible techniques have undergone validation for the assessment of endothelial function in clinical settings [26].

Mid-regional proadrenomedullin has found utility primarily in patients with developing sepsis [40,41,43]. This parameter is exceptionally sensitive and specific compared to others, and importantly, an increase in MR-proADM is observed earlier than an increase in C-reactive protein (CRP) or procalcitonin (PCT) [40,43,44]. ADM functions as a regulatory component in the immune system, influencing complement activity and contributing to increased serum levels during sepsis. Despite its pivotal role, detecting ADM directly in blood samples poses significant challenges due to its rapid degradation. To circumvent this issue, MR-proADM has emerged as a valuable alternative, providing a reliable reflection of serum ADM levels [45]. Among the array of biomarkers currently under investigation, including presepsin and interleukin-6, MR-proADM is emerging as a promising tool in the clinical management of hospitalized patients with sepsis and septic shock. This is underscored by its documented vasodilatory and antibacterial properties, which are increasingly corroborated in the literature [15,40].

## 7. Viral Diseases

ADM and MR-proADM were used mostly in critically ill patients with COVID-19. Primarily, it has been used to assess the risk of mortality [45]. Among the advantages of ADM, we can include its sensitivity to early elevation and its high diagnostic accuracy [45]. This biomarker demonstrates a rapid increase in cases of severe viral or bacterial infections, serving as an indicator of their gravity and assisting healthcare professionals in formulating treatment strategies and determining the most suitable care environment for patients.

## 8. COVID-19

COVID-19, induced by the SARS-CoV-2 virus, which belongs to the *Coronaviridae* family, predominantly affects the human respiratory system. Its clinical manifestations are diverse, ranging from minimally symptomatic cases to severe, life-threatening conditions, including mortality [10]. The virus replicates within host cells, eliciting an inflammatory response that ultimately results in multiple organ failure. The clinical course of the disease is difficult to predict, prompting the search for biomarkers that can identify individuals at higher risk of severe outcomes. MR-proADM has been utilized for this purpose [10,45,46].

Research has shown that patients with COVID-19 had elevated levels of MR-proADM [10]. The levels of markers in plasma were measured during the collection of biological material upon patient admission. The relationship between plasma MR-proADM levels and the severity of the disease was assessed, with severity defined as the necessity for mechanical ventilation and in-hospital mortality. Under physiological conditions, MR-proADM levels are typically very low [10,46]. Lo Sasso [10] emphasized that 72% of patients exhibited significantly elevated concentrations of MR-proADM in plasma, with a median concentration of 0.93 nmol/L (IQR, 0.58–1.09) [10].

Furthermore, it was found that MR-proADM levels correlate with biochemical parameters indicative of the inflammatory state. Notably, a statistically significant correlation was observed between MR-proADM and C-reactive protein (CRP) values. An MR-proADM level of 1.73 nmol/L was identified as the optimal cutoff for predicting mortality, demonstrating a sensitivity of 90% and a specificity of 95% [10].

Moreover, this level was significantly higher in individuals who died during hospitalization compared to those who survived [10,46]. MR-proADM has been demonstrated to be a more sensitive indicator compared to CRP and IL-6 [45]. It has been demonstrated that MR-proADM is associated with other biochemical markers of inflammation, such as CRP, LDH, IL-6, PCT, and hs-TnT. Additionally, it has shown a negative correlation with vitamin D and the percentage of lymphocytes [10].

The assessment of MR-proADM levels has also found application in patients admitted to medical intensive care units, as well as in individuals with community-acquired pneumonia [10,38]. It should be emphasized that community-acquired pneumonia can be caused by bacterial or viral infections. The most common bacterial pathogens include *Streptococcus pneumoniae*, *Haemophilus influenzae*, *Moraxella catarrhalis*, *Staphylococcus aureus*, Group B *Streptococcus*, aerobic Gram-negative bacteria such as *Enterobacteriaceae* (e.g., *Escherichia coli*), and atypical pathogens such as *Mycoplasma pneumoniae*, *Chlamydophila pneumoniae*, and *Legionella pneumophila*. Viral etiologies contribute to approximately 30 to 50% of pneumonia cases, with the most common viruses being coronaviruses, influenza viruses (A and B), rhinoviruses, parainfluenza virus, adenoviruses, RSV, human bocavirus, and metapneumovirus [38,47,48].

## 9. Influenza

Influenza, caused by influenza viruses from the *Orthomyxoviridae* family, is an acute respiratory illness. It is characterized by a sudden onset of fever, cough, sore throat, muscle aches, and fatigue, and can lead to severe complications such as pneumonia, especially in vulnerable populations like the elderly and immunocompromised individuals. ADM has been applied not only in assessing the course of SARS-CoV-2 infection but also in various other clinical contexts. As a biomarker, ADM has been utilized in evaluating the progression of influenza. Biomarker levels were determined at the time of admission. The optimal cutoff for severity, defined as the necessity for intensive care unit (ICU) admission, was established at MR-proADM levels of 1.09 nmol/L, with a sensitivity of 73.53% and a specificity of 96% [38]. Initial MR-proADM levels were found to be effective in predicting unfavorable outcomes, as well as the risk of ICU admission and mortality in patients with pneumonia caused by the influenza A virus [38,49,50,51]. In patients with influenza virus pneumonia, the initial levels of MR-proADM, ferritin, CRP, and PCT serve as valuable indicators for predicting adverse outcomes, risk of ICU admission, and mortality. Remarkably, MR-proADM demonstrates the greatest predictive power for survival [38,51].

## 10. Dengue Fever

Dengue fever is a viral disease caused by the dengue virus, a member of the *Flaviviridae* family [52,53,54]. The virus is primarily transmitted by Aedes mosquitoes, especially Aedes aegypti and Aedes albopictus [53,54]. Dengue fever is prevalent in tropical and subtropical regions worldwide, including Latin America, Southeast Asia, Africa, and the Pacific Islands [54].

Symptoms of dengue fever include sudden high fever, severe headaches, pain behind the eyes, muscle and joint pain, rash, and bleeding (e.g., from the nose or gums). In severe cases, it can progress to dengue hemorrhagic fever, characterized by hemorrhagic manifestations and dengue shock syndrome, a life-threatening condition requiring immediate medical attention [52,53,55].

Currently, there is no specific antiviral treatment for dengue fever, and management is mainly symptomatic, including fluid replacement, antipyretics, and pain relief. Severe cases may require hospitalization and intensive medical care [52,53,55].

Sources report that adrenomedullin has been used as a marker of disease severity [56]. Adrenomedullin is classified as a vasoactive hormone, influencing endothelial permeability and vascular tone and playing a role in maintaining proper hydration and electrolyte balance. It is suggested that, as a peptide, it impacts the pathogenesis of dengue [56]. Notably, elevated ADM levels have been observed not only in dengue hemorrhagic fever (DHF) but also in dengue shock syndrome (DSS) [56]. Increased ADM values have also been correlated with low albumin levels and increased pleural effusion [56].

Adrenomedullin (ADM) is significant in dengue fever for several reasons. It serves as a biomarker for disease severity, aiding in the assessment of patient status and outcome prediction. As a vasoactive hormone, ADM affects vascular permeability and tone, which are crucial during inflammatory responses in dengue. Elevated levels of ADM are notably found in both dengue hemorrhagic fever (DHF) and dengue shock syndrome (DSS), indicating its role in severe complications. Additionally, increased ADM concentrations correlate with low albumin levels and greater pleural effusion, reflecting its association with organ dysfunction.

Moreover, the use of ADM as a biomarker allows for the prediction of clinical outcomes and the implementation of appropriate treatments, making therapy more personalized. These factors highlight the potential of adrenomedullin in enhancing the understanding of dengue pathogenesis and its clinical implications [56].

## 11. Role of Adrenomedullin in the Central Nervous System

According to published studies, the application of adrenomedullin is not limited to determining therapeutic opportunities in bacterial and viral infections. Articles report that adrenomedullin is a significant factor that can provide insights into the state of the central nervous system (CNS).The gene encoding ADM exhibits widespread expression throughout the CNS, with various roles proposed for AM in the brain [31]. It has been observed that physiological levels of adrenomedullin increase with age and that pathological increases may suggest neurodegenerative changes. These findings have been consistently obtained in studies on mice and post mortem examinations of human brains [57]. Adrenomedullin has also been linked to neurodegenerative and neurological diseases (especially migraines), as well as increased sensitivity to pain stimuli [15,58].

In studies conducted on mice, an intriguing relationship has been observed. Animals in which the expression of the ADM gene in the brain was eliminated through genetic engineering exhibited coordination problems and increased sensitivity to hypoxia, suggesting a neuroprotective function. Furthermore, behavioral analysis documented changes in the animals’ behavior, such as hyperactivity and excessive anxiety, compared to their healthy littermates [31].

## 12. Future Prospects for Adrenomedullin: Applications in Viral Meningitis and Beyond

Based on the available literature, there is hope for the use of adrenomedullin in detecting other viral diseases. There is a need to investigate whether adrenomedullin could be useful in other infections caused by viruses from the *Flaviviridae*, *Coronaviridae*, and *Orthomyxoviridae* families.

There is considerable hope regarding the affinity of ADM and mr-proADM for the brain and CNS. Given their effective response in detecting infections of both viral and bacterial etiology, it is anticipated that these markers could be useful in identifying CNS infections, whether viral or bacterial. However, this is a matter that requires further investigation. If our assumptions are correct, this could lead to personalized treatment and the identification of patient groups with particularly severe courses or those at risk of complications, necessitating special medical attention.

One particular form of encephalitis stands out as a future therapeutic possibility for the routine use of adrenomedullin in diagnostics: tick-borne encephalitis.

Tick-borne encephalitis is a disease caused by a virus from the *Flaviviridae* family, for which there is no cure and which can lead to permanent neurological sequelae and complications [59,60,61,62,63,64]. Therefore, further research on the utility of mr-proADM in exploring therapeutic opportunities for patients is essential.

The utility of ADM in viral infections is further highlighted by its potential to differentiate between viral and bacterial etiologies, particularly in infections of the central nervous system (CNS). The accurate identification of viral pathogens could help avoid unnecessary antibiotic use, reducing the risk of antibiotic resistance. This is especially relevant in cases of viral meningitis or encephalitis, where early distinction between viral and bacterial causes is essential for appropriate treatment. By improving the precision of diagnoses, ADM could help clinicians avoid the inappropriate use of antibiotics in viral infections, thereby enhancing antimicrobial stewardship efforts.

Beyond the diseases where ADM has already demonstrated clinical relevance, such as dengue and COVID-19, there is growing interest in its application to other viral infections. Further investigation into the role of ADM could potentially benefit the study of various viral infections, such as those caused by viruses from the Flaviviridae family (Zika virus, yellow fever) as well as others like Ebola and HIV. In these infections, where vascular involvement and systemic inflammation play a significant role in disease progression, ADM could serve as a biomarker for both early detection and long-term management, guiding therapeutic strategies and improving patient outcomes.

Adrenomedullin (ADM) has garnered attention for its diverse biological roles, particularly in modulating vascular permeability, inflammation, and organ function. This peptide hormone has shown promise as a biomarker in assessing disease severity across various viral infections, owing to its involvement in endothelial function and immune regulation. Its potential as a diagnostic and prognostic tool in viral diseases extends beyond its current applications, suggesting that it may be valuable in a broader spectrum of infections.

## 13. Conclusions

It is beyond doubt that adrenomedullin has attracted the attention of scientists due to its multifunctionality and usefulness as a hypothetical biomarker in conditions of both bacterial and viral etiology. Mr-proADM possesses many advantages, rendering it an ideal candidate as a biomarker to be measured in numerous disorders. This could contribute to refining diagnostic and therapeutic methodologies, thereby advancing healthcare provision and improving treatment efficacy. It is worth emphasizing that there still remain many unknowns regarding adrenomedullin and mr-proADM, and that we have yet to fully harness the potential encapsulated by this substance. Therefore, there is a pressing need for further research aimed at better understanding the functions of adrenomedullin in the body and its optimal utilization in daily clinical practice.
